# Novel α, β-Unsaturated Sophoridinic Derivatives: Design, Synthesis, Molecular Docking and Anti-Cancer Activities

**DOI:** 10.3390/molecules22111967

**Published:** 2017-11-14

**Authors:** Yiming Xu, Lichuan Wu, Hang Dai, Mingyan Gao, Haroon Ur Rashid, Haodong Wang, Peng Xie, Xu Liu, Jun Jiang, Lisheng Wang

**Affiliations:** 1 School of Chemistry and Chemical Engineering, Guangxi University, Nanning 530004, China; xuyimingcorey@163.com (Y.X.); haroongold@gmail.com (H.U.R.); 18369903816@163.com (H.W.); xiep@gxu.edu.cn (P.X.); wendaoliuxu@163.com (X.L.); 2 Medicinal College, Guangxi University, Nanning 530004, China; wulichuan@126.com; 3 State Key Laboratory for Chemistry and Molecular Engineering of Medicinal Resources, Guangxi Normal University; Guilin 541000, China; 4 College of Pharmacy, Guangxi University of Chinese Medicine, Nanning 530004, China; hangdai5180@163.com (H.D.); GMYjiushizhu@163.com (M.G.); 5 Department of Chemistry, Sarhad University of Science & Information Technology, Peshawar, Khyber Pakhtunkhwa 25120, Pakistan

**Keywords:** sophoridine, chalcone, derivatives, anticancer activity, molecular docking

## Abstract

Using sophoridine **1** and chalcone **3** as the lead compounds, a series of novel α, β-unsaturated sophoridinic derivatives were designed, synthesized, and evaluated for their in vitro cytotoxicity. Structure-activity relationship (SAR) analysis indicated that introduction of α, β-unsaturated ketone moiety and heterocyclic group might significantly enhance anticancer activity. Among the compounds, **2f** and **2m** exhibited potential effects against HepG-2 and CNE-2 human cancer cell lines. Furthermore, molecular docking studies were performed to understand possible docking sites of the molecules on the target proteins and the mode of binding. This work provides a theoretical basis for structural optimizations and exploring anticancer pathways of this kind of compound.

## 1. Introduction

Traditional herbs contain numerous biologically active natural compounds. Such compounds have been reported to possess remarkable therapeutic value with slight harmful effects. Their therapeutic efficacy offers a useful platform for developing new standard drugs. Sophoridine is a quinolizidine monomeric alkaloid isolated from traditional Chinese herbs. It is found in leaves and stem of Leguminous plants *Sophora alopecuroides* L., *Euchresta japonica* Benth., and the roots of *Sophora alopecuroides* Ait. Evidence suggests that sophoridine has remarkable pharmacological effects including anti-inflammatory, anti-viral, and anti-cancer effects. Sophoridine has been used to treat malignant tumors for decades in china [1,2,3,4,5,6,7]. It exhibits the potential therapeutic efficacy against gastric, colon and lung cancer. The mechanism of action of sophoridine is to inhibit DNA topoisomerase I (Topo I) activity and induce cell cycle arrest at the G0/G1 phase and then cause apoptotic cell death [8]. Compared to other DNA topo I inhibitors, such as 10-hydroxycamptothecin (HCPT) and topotecan, sophoridine has many advantages such as a special chemical scaffold, flexibility structure, high solubility, and good safety profiles [9]. However, the moderate antitumor activities of sophoridine limit its use as a drug for clinical application, suggesting that it is an ideal lead compound for further modifications and optimizations. 

Natural chalcones belong to flavonoids and are widely distributed in natural plants. They display various pharmacological activities including the anticarcinogenic properties of xanthohumol [10] isolated from the hop cones, the chemopreventive effect of isoliquiritigenin [11] isolated from the *Glycyrrhiza uralensis*, and the anti-adipogenic effect of butein [12] isolated from the stems of *Rhus verniciflua*. Synthetic chalcone derivatives have been reported to possess antitumour [13], antimicrobial [14] and anti-inflammatory activities [15]. These activities are mainly due to their α, β-unsaturated ketone moiety and various substituents introduced to the aryl rings [16]. 

Moreover, some heterocyclic compounds containing N, S, and O are common active drugs such as Furazolidone, Dolasteron, and Melotonin. It is reported that these drugs possess anti-inflammatory, antibacterial, antitumor, anticancer, and other important physiological and pharmaceutical activity [17,18,19,20,21,22,23].

**Figure 1:**
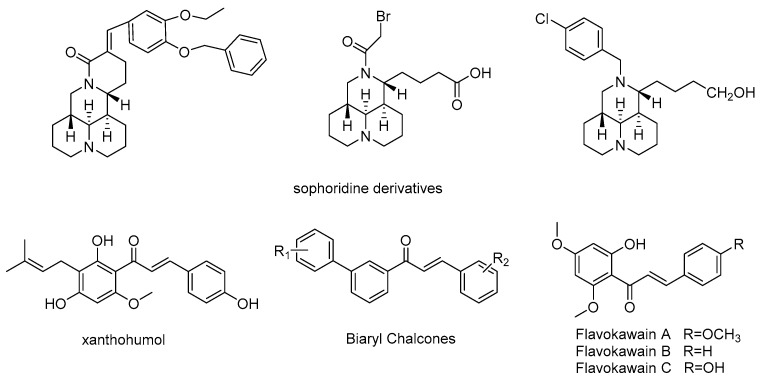
Sophoridine and chalcones derivatives bearing anticancer activities.

There are strong evidences that chalcones and sophoridine derivatives exhibit potent anticancer activities [24,25,26,27] (Figure 1). Based on the above suggestion, we hypothesize that building α, β-unsaturated ketone moiety with aryl ring into the structure of sophoridine might be beneficial for improving antitumor activity and inducing toxicity.

**Scheme 1:**
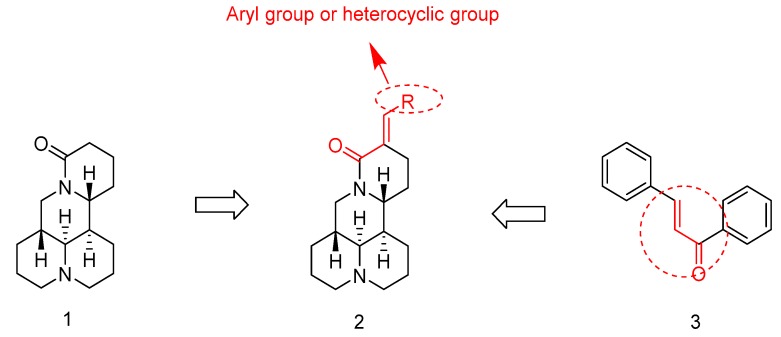
The modification strategy of new scaffold based on the structures of sophoridine **1**, chalcone **3**.

Furthermore, many heterocyclic groups were also introduced into the structure of α, β-unsaturated ketone moiety. On the basis of this strategy, 19 novel α, β-unsaturated sophoridine derivatives were subsequently designed, synthesized and evaluated for their cytotoxicity in the present work (Scheme 1).

## 2. Results

### 2.1. Chemistry

**Scheme 2:**
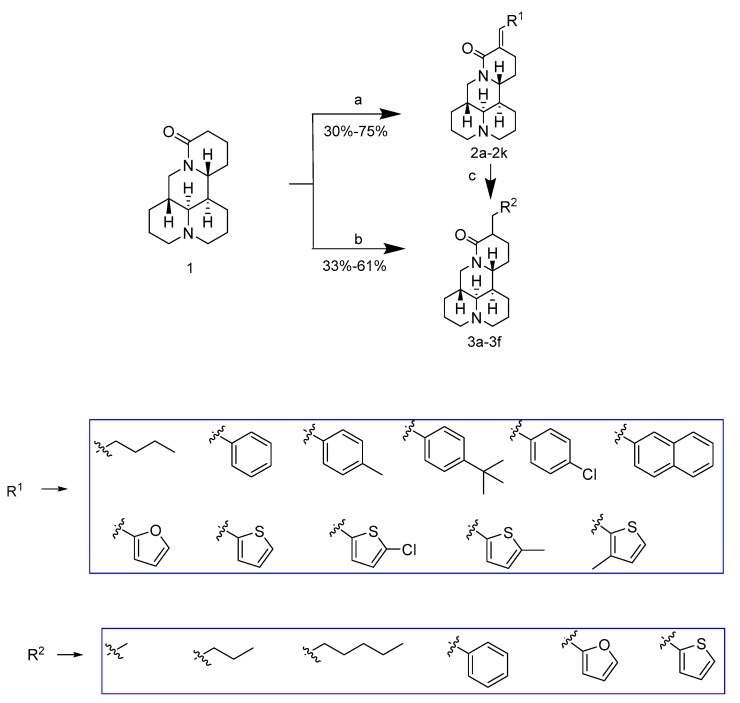
Synthesis routes of sophoridinic derivatives. Reagents and conditions: (a) R^1^CHO, NaH, THF, 80 °C, 5~6 h; (b) R^2^CH_2_Br, LDA, THF, r.t., 4~5 h; (c) H_2_, Pd, MeOH, r.t., 1 bar, 3 h.

Seventeen α, β-unsaturated sophoridinic analogs were synthesized using commercially available **1** as the starting material as depicted in Scheme 2. The carbanion intermediate was obtained via the dehydrogenation of 1 under the electronic absorption effect of carbonyl, and then reacted with aldehyde and dehydrated to prepare **2a**–**2k** in alkaline condition [28]. **3a**–**3f** were directly synthesized in 45–68% yields by substitution of intermediate with halide. The desired products **3a**–**3f** could also be prepared via reduction under hydrogen with Pd in methanol solution for 3 h [29].

### 2.2. SAR Analysis for the Antiproliferative Activity

The 17 newly synthesized compounds were evaluated for their anticancer activities in human HepG-2 and CNE-2 cell lines using MTT assay through in vitro investigations. As shown in Table 1, SAR analysis was first focused on the substituent at the 14 position of sophoridine.

A group of aliphatic alkyl including ethyl, butyl, pentyl, and hexyl were firstly introduced at the 14 position using two methods in which four 14-substituted sophoridinic analogues (**2a**, **3a**, **3b**, **3c**) were made and tested. The results showed that presence of α, β-unsaturated ketone moiety in these compounds and the length of chain could influence the cytotoxic activity. We speculated that the groups would enhance the stability and lipophilicity owing to the changing of the electronic environment which in turn improves the activity. Similarly, four compounds **2b**, **2c**, **2d**, **3d** possessing substituted aromatics at the same position were also prepared and evaluated. The results also showed that α, β-unsaturated ketone could enhance the activity, while **2b** afforded better antiproliferative activities than **3d**. More importantly, **2b**–**2d** afforded moderate antiproliferative activities. It seemed that the improved anticancer activities of compounds **2b**–**2d** were consistent with their relatively Clog P values calculated by ChemBioOffice software (version 12.0). Then, naphthyl and *tert*-butyl phenyl with high lipophilicity were introduced at the 14 position aiming at enhancing the Clog P values, thereby enhancing the activity against cancer, with which new corresponding compounds (**2e**, **2f**) were made and examined. As anticipated, the compound **2e** displayed higher potency than that of sophoridine. 

Since heterocyclic group is crucial for the anticancer activity in many natural medicines, therefore, this group was introduced at position 14 to generate corresponding new derivatives (**2g**–**2k**, **3e**–**3f**). SAR results suggested that the heterocyclic ring with specific substituent could significantly influence the anticancer activities and activities of compounds **2g** and **3e** showed that α, β-unsaturated ketone moiety plays an important role in their improvement. Among the compounds, sophoridinic analogues **2k** exhibited potent activity against HepG2 and SNE-2 cancer with IC_50_ values ranging from 25 to 33 μM.

We speculated that the activities of new sophoridine compounds could be improved by increasing interactions between π–π stacking of the aromatic nucleus and hydrogen bonds of the heterocyclic group of target DNA base pairs and protein residues. Furthermore, the variety and position of substituents on the ring significantly affect the electronic environment of the new sophoridine derivatives. These changes will influence bioavailability, susceptibility to metabolism, and the pharmacological profile of the resulting analogues. We envisioned that chemical modification would further improve the activity of these resulting sophoridine compounds.

Finally, compounds **2e** and **2k** bearing potent anticancer effects as well as reasonable Clog P values were selected as representatives for further investigation.

### 2.3. Cell Cycle Analysis

**Figure 2:**
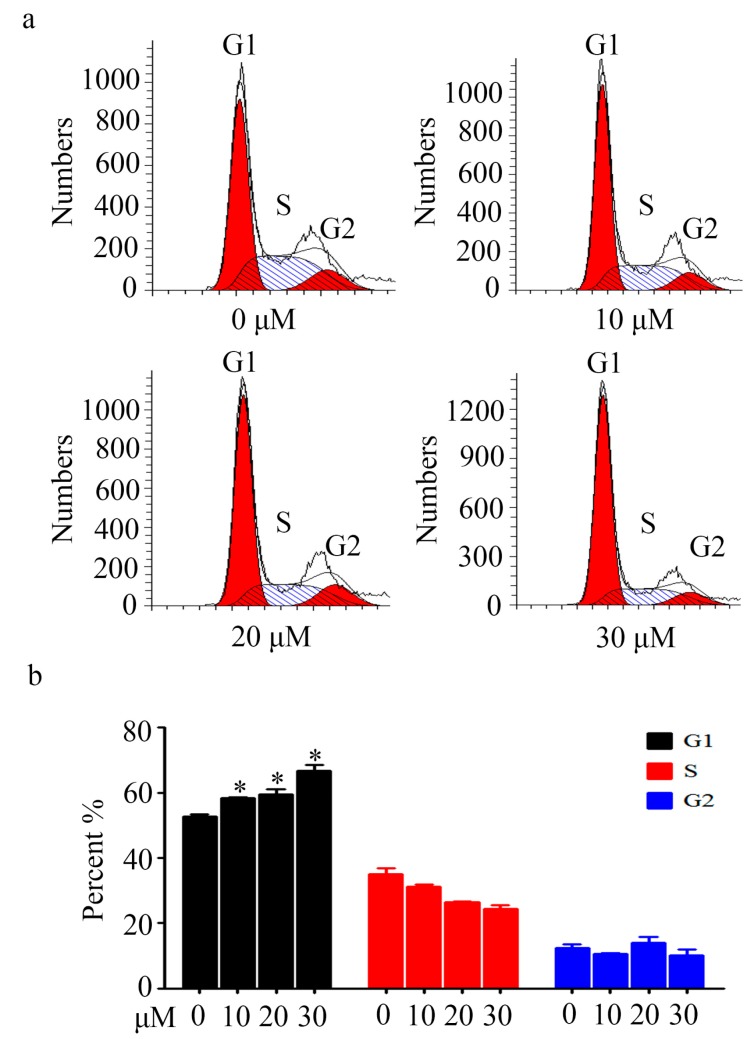
Cell cycle analysis of **2k**. (**a**) HepG2 cells were incubated without (control) or with compound at different concentrations (10, 20, 30 μmol/L) for 24 h-and then analyzed for their cell cycle distribution using flow cytometry; (**b**) The percentage of each population was shown as mean ± SD. Data is the representative of three independent experiments. * *p* < 0.05.

We extended our work to the primary mechanism investigation using compound **2k**. Flow cytometric analysis in the HepG2 cells was carried out. As shown in Figure 2, compound **2k** arrested HepG2 cells at the G1 phase in a dose-dependent manner, indicating compound **2k** inhibited HepG2 cell growth through cell cycle arrest.

### 2.4. Molecular Docking

**Figure 3:**
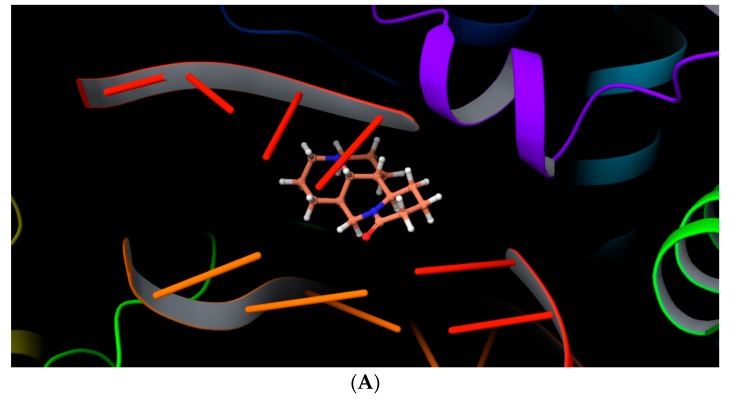

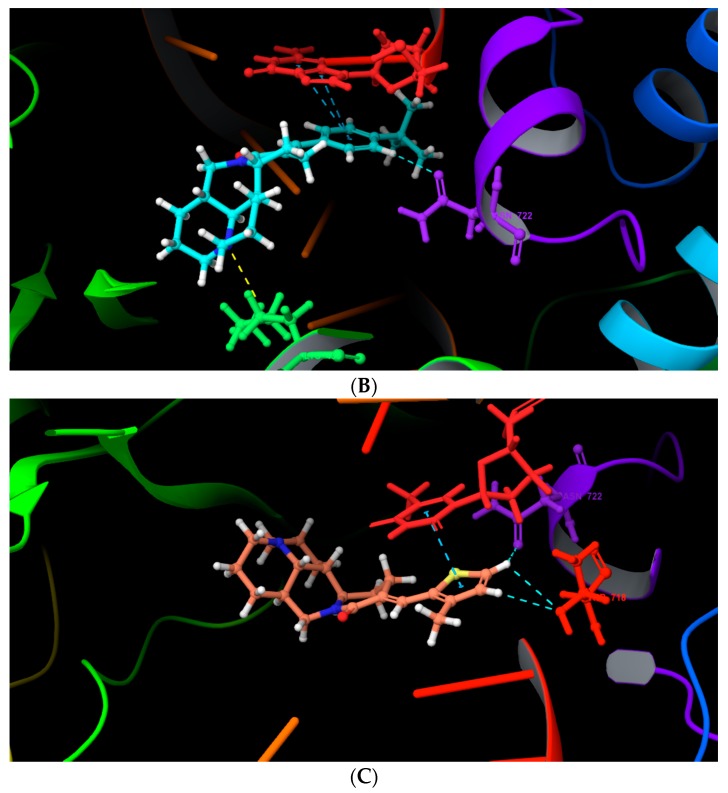
Molecular docking models of sophoridine (**A**); **2e** (**B**); **2k** (**C**) in the active site of DNA–Topo I complex (PDB ID: 1T8I).

In order to better understand the potency of α, β-unsaturated derivatives for further SAR study, molecular docking studies with human DNA-Topo I complex were also performed. All calculations were performed using Glide model from Schrödinger software. The crystal structure of DNA-Topo I complex with Camptothecin (PDB ID: 1T8I) was obtained from the protein data bank (PDB). Molecular docking studies of the compounds were performed to predict the binding affinity of our newly synthesized derivatives into the binding site of DNA-Topo I complex, which contribute to rationalize the obtained biological results and their mechanism of action. Additionally, molecular docking studies helped us to understand various interactions between the ligands and the receptor in detail. Human topoisomerase I cleaves a single DNA strand. Topo I poisons can bind to the covalent Topo I-DNA complex through various interactions, resulting in double strand breaks and ultimately apoptotic cell death. As seen in Figure 3, the primary interactions of the sophoridine structure with DNA-Topo I complex are through the formation of force like Van der Waals, hydrophobic bond with residues of protein. Moreover, interaction between compound **2e** and protein could also occur through hydrogen bonds from ASN722 in protein with *tert*-butyl group, while the ASN722 and THR718 are hydrogen bonded to the thienyl group of compound **2k**. Besides, the docking results revealed that the introduction of groups in these compounds contribute to form a stable complex in DNA-Topo I active site through π–π stacking interaction with purine ring of DNA. These interactions revealed the importance of both protein and DNA for binding and the subsequent inhibitory capacity.

The resulting docking model gives minimum relative binding energy for **2e** and **2k** as −44.39 kcal·mol^−1^ and −44.97 kcal·mol^−1^, respectively, compared to that of sophoridine (−28.54 kcal·mol^−1^), also indicating that phenyl and heterocyclic groups introduced at 14 position of sophoridine might be beneficial for the anticancer activity. The binding model does not provide any clue to explain the action of α, β-unsaturated ketone moiety. Furthermore, it fails to explain why the activity of **2e** is better than **2b** and that of **2k** is better than **2j**. We speculated the variety and position of substituents on the heterocyclic group and that α, β-unsaturated ketone could influence the density and distribution of electron cloud, then altering the binding affinity. Further studies on the mechanism of action are currently in progress in our laboratory.

## 3. Materials and Methods

### 3.1. Instrumentation

All the compounds were characterized with ^1^H-NMR, ^13^C-NMR, and MS. ^1^H and ^13^C nuclear magnetic resonance (NMR) data were recorded on Bruker Avance 600 (600 MHz) spectrometer (Brucker, Inc., Silberstreifen, Rheinstetten, Germany) with CDCl_3_ as solvent and tetramethylsilane (TMS) as an internal standard. Coupling constants (*J*) were in hertz (Hz), and signals were designated as follows: s, singlet; d, doublet; t, triplet; m, multiplet; br, broad singlet, etc. Mass spectra were obtained from a ThermoFisher LCQ Fleet (ESI). Melting points were determined in open capillary tubes on X-4 melting point apparatus without correction. The optical density was measured at the 490 nm wavelength on an enzyme-linked immunosorbent assay microplate reader (Fisher Scientific International, Inc. Hampton, NH, USA). The progress of reactions was monitored by Thin Layer Chromatography (TLC) analysis (glass sheets coated with silica gel) (Yantai, China). Detections were done by UV light (254 nm) illumination and/or treatment with Bismuth potassium iodide solution. The products were purified by flash column chromatography equipped with commercial silica gel (300–400 mesh).

### 3.2. Materials

Sophoridine (99%) was purchased from Shanxi Undersun Biomedtech Co., Ltd. (Xinke Plaza, No.18 Keji Road, Xi’an, China). All other chemicals and reagents used in the experiments were of analytical grade and obtained from Sinopharm Chemical Reagent Co., Ltd. (52 Ning Bo Road, Shanghai, China)

### 3.3. Experimental Procedures and Characterization

#### 3.3.1. Synthesis of Compounds **2a**–**2k**

Anhydrous tetrahydrofuran (50 mL) was added into a round-bottomed flask (100 mL) containing sophoridine (0.005 mol) and sodium hydride (0.1 mol). The solution was stirred, and aldehyde (0.02 mol) was added at 35–40 °C. The solution was then refluxed for 8 h. After cooling to room temperature, the mixture was treated with hydrochloric acid (5%, 20 mL) to hydrolyze the excess sodium hydride and then extracted with chloroform (3 × 20 mL). The combined organic layer was concentrated, and the residue was purified in a reverse-phase silica gel column (CH_2_Cl_2_:MeOH = 20:1, *v*/*v*) to give compounds **2a**–**2k**.

#### 3.3.2. Synthesis of Compounds **3a**–**3f**

To a round-bottomed flask (100 mL) containing LDA (3 mL) and sophoridine (5 mmol, 1.24 g) dissolved in anhydrous tetrahydrofuran (50 mL) was added dropwise at 0 °C and stirred for 40 min, then alkyl halide (0.02 mol) was added and reacted at room temperature for 5 h. The mixture was treated with hydrochloric acid (5%, 20 mL) to neutralize and then extracted with chloroform (3 × 20 mL). The combined organic layer was dried with anhydrous sodium sulfate and then concentrated. The residue was purified by silica gel column chromatography using CH_3_COOEt/CHCl_3_ (50:1, *v*/*v*) as eluent to give the compounds **3a**–**3f**.

**2a** Yield: 30%; Brown oil; ^1^H-NMR (600 MHz, Chloroform-*d*) δ 6.74 (m, 1H), 3.90 (m, 1H), 3.14 (d, *J* = 12.8 Hz, 1H), 2.90 (m, 3H), 2.58–2.49 (m, 1H), 2.32–2.24 (m, 1H), 2.22–2.08 (m, 5H), 1.99 (m, 3H), 1.90 (m, 1H), 1.80–1.64 (m, 4H), 1.64–1.48 (m, 3H), 1.47–1.35 (m, 5H), 0.90 (m, 3H). ^13^C-NMR (151 MHz, Chloroform-*d*) δ 165.13, 137.26, 128.75, 63.07, 55.79, 55.49, 50.20, 47.74, 40.06, 31.28, 30.89, 30.00, 27.51, 27.22, 23.39, 22.40, 21.91, 21.58, 21.46, 13.89. MS (ESI) *m*/*z*: 317.363 [M + H]^+^.

**2b** Yield: 64%; Yellow oil; ^1^H-NMR (600 MHz, Chloroform-*d*) δ 7.74 (d, *J* = 1.7 Hz, 1H), 7.41–7.36 (m, 2H), 7.36–7.33 (m, 2H), 7.32–7.30 (m, 1H), 4.53 (m, 1H), 3.99 (m, 1H), 3.25 (t, *J* = 12.7 Hz, 1H), 2.95–2.80 (m, 3H), 2.51 (m, 1H), 2.17 (d, *J* = 3.0 Hz, 1H), 2.12 (m, 1H), 2.01 (m, 2H), 1.90 (m, 1H), 1.85–11.26 (m, 10H). ^13^C-NMR (151 MHz, Chloroform-*d*) δ 164.83, 136.35, 134.40, 130.96, 129.52 (2), 128.21 (2), 127.68, 63.80, 57.26, 57.22, 52.85, 42.77, 42.64, 35.64, 27.77, 26.40, 25.90, 23.10, 21.18, 20.79. MS (ESI) *m*/*z*: 337.622 [M + H]^+^.

**2c** Yield: 40%; Yellow oil; ^1^H-NMR (600 MHz, Chloroform-*d*) δ 7.72 (s, 1H), 7.35–7.31 (m, 2H), 6.95–6.90 (m, 2H), 3.85 (s, 3H), 3.61 (m, 1H), 3.56–3.47 (m, 1H), 3.36 (t, *J* = 12.8 Hz, 1H), 2.99–2.86 (m, 2H), 2.60 (m, 1H), 2.29–2.16 (m, 1H), 2.14–2.04 (m, 2H), 1.98–1.86 (m, 2H), 1.75 (d, *J* = 13.4 Hz, 1H), 1.61–1.46 (m, 4H), 1.41–1.23 (m, 4H), 1.15 (m, 1H), 0.95–0.83 (m, 1H). ^13^C-NMR (151 MHz, Chloroform-*d*) δ 165.34, 159.28, 133.92, 131.21 (2), 128.78, 128.28, 113.74 (2), 63.42, 55.98, 55.36, 55.30, 50.50, 48.11, 40.09, 31.77, 29.70, 27.39, 23.78, 23.63, 21.52. MS (ESI) *m*/*z*: 367.610 [M + H]^+^.

**2d** Yield: 55%, m.p.: 150.1–151.6 °C; White solid; ^1^H-NMR (600 MHz, Chloroform-*d*) δ 8.01 (d, *J* = 8.1 Hz, 2H), 7.39 (s, 1H), 7.27–7.24 (m, 1H), 7.17–7.14 (m, 1H), 3.78–3.60 (m, 2H), 3.60–3.48 (m, 2H), 3.40–3.31 (m, 1H), 3.26 (m, 1H), 2.76–2.62 (m, 2H), 2.53 (m, 1H), 2.24 (m, 2H), 2.08–2.01 (m, 1H), 1.95 (d, *J* = 13.0 Hz, 2H), 1.84–1.63 (m, 2H), 1.54–1.37 (m, 1H), 1.24 (m,3H), 0.95–0.82 (m, 2H). ^13^C-NMR (151 MHz, Chloroform-*d*) δ 164.88, 134.84, 132.74, 131.11, 130.56 (2), 128.50 (2), 128.23, 63.92, 56.85, 53.00, 45.39, 36.10, 31.91, 29.70, 29.33, 28.63, 27.22, 22.70, 20.66, 14.13. MS (ESI) *m*/*z*: 371.517 [M + H]^+^.

**2e** Yield: 62%, m.p.: 65.1–65.7 °C; White solid; ^1^H-NMR (600 MHz, Chloroform-*d*) δ 7.74 (d, *J* = 1.7 Hz, 1H), 7.43–7.38 (m, 2H), 7.31 (d, *J* = 8.2 Hz, 2H), 3.61 (dd, *J* = 13.7, 5.0 Hz, 1H), 3.51 (m, 1H), 3.35 (dd, *J* = 13.7, 11.7 Hz, 1H), 2.95–2.87 (m, 2H), 2.84–2.77 (m, 1H), 2.60 (m, 1H), 2.23–2.12 (m, 2H), 2.12–1.79 (m, 5H), 1.73 (m, 1H), 1.65–1.59 (m, 1H), 1.59–1.42 (m, 4H), 1.34 (s, 9H), 1.33–1.22 (m, 1H), 1.13 (m, 1H). ^13^C-NMR (151 MHz, Chloroform-*d*) δ 165.23, 150.96, 134.04, 133.35, 129.49 (2), 129.46, 125.18 (2), 63.55, 56.05, 55.38, 50.62, 48.18, 40.16, 34.67, 31.88, 31.26 (3), 30.07, 27.42, 23.96, 23.64, 21.60, 21.52. MS (ESI) *m*/*z*: 393.626 [M + H]^+^.

**2f** Yield: 30%, m.p.: 151.7–153.6 °C; White solid; ^1^H-NMR (600 MHz, Chloroform-d) δ 8.92 (d, *J* = 8.5 Hz, 1H), 8.11 (d, *J* = 7.1 Hz, 1H), 7.92 (d, *J* = 8.1 Hz, 1H), 7.87 (m, 2H), 7.54–7.44 (m, 3H), 3.64 (m, 2H), 3.42–3.23 (m, 2H), 2.77–2.62 (m, 2H), 2.52–2.40 (m, 2H), 2.25–2.15 (m, 1H), 2.14–1.16 (m, 13H). ^13^C-NMR (151 MHz, Chloroform-*d*) δ 165.03, 133.87, 133.15, 131.23, 128.26, 126.74, 126.59, 126.26, 126.05, 125.66, 125.08, 124.94, 124.77, 60.42, 56.16, 54.72, 50.80, 47.58, 39.42, 29.58, 29.33, 27.32, 27.23, 23.50, 20.66, 14.13. MS (ESI) *m*/*z*: 387.514 [M + H]^+^.

**2g** Yield: 73%, m.p.: 96.1–98.3 °C; White solid; ^1^H-NMR (600 MHz, Chloroform-*d*) δ 7.54–7.45 (m, 2H), 6.53 (d, *J* = 3.4 Hz, 1H), 6.47 (m, 1H), 3.61 (m, 1H), 3.55–3.50 (m, 1H), 3.33 (m, 1H), 3.16 (m, 1H), 2.91 (m, 1H), 2.82 (m, 1H), 2.75–2.67 (m, 1H), 2.26–1.25 (m, 14H), 1.13 (m, 1H). ^13^C-NMR (151 MHz, Chloroform-*d*) δ 164.98, 152.59, 143.41, 126.94, 121.10, 114.03, 111.74, 63.50, 56.03, 55.45, 50.61, 48.09, 40.01, 31.99, 29.99, 26.87, 23.88, 23.54, 21.57, 21.53. MS (ESI) *m*/*z*: 327.364 [M + H]^+^.

**2h** Yield: 75%, m.p.: 155.6–158.2 °C; White solid; ^1^H-NMR (600 MHz, Chloroform-*d*) δ 7.93 (d, *J* = 2.0 Hz, 1H), 7.43 (d, *J* = 5.1 Hz, 1H), 7.25 (d, *J* = 3.6 Hz, 1H), 7.10 (dd, *J* = 5.1, 3.6 Hz, 1H), 4.49 (dd, *J* = 12.7, 4.5 Hz, 1H), 4.00 (m, 1H), 3.24 (t, *J* = 12.7 Hz, 1H), 2.97 (m, 1H), 2.90–2.79 (m, 2H), 2.64 (m, 1H), 2.26–2.09 (m, 2H), 2.05–1.25 (m, 13H). ^13^C-NMR (151 MHz, Chloroform-*d*) δ 164.93, 139.14, 131.41, 129.47, 128.23, 127.65, 127.32, 61.61, 55.59, 54.95, 50.77, 49.03, 47.52, 39.17, 29.39, 26.55, 23.70, 21.76, 21.59, 20.56. MS (ESI) *m*/*z*: 343.367 [M + H]^+^.

**2i** Yield: 43%, m.p.: 131.3–133.0 °C; Yellow solid; ^1^H-NMR (600 MHz, Chloroform-*d*) δ 7.66 (d, *J* = 4.1 Hz, 1H), 7.60 (d, *J* = 4.1 Hz, 1H), 6.99 (d, *J* = 1.4 Hz, 1H), 4.07 (m, 1H), 3.53–3.46 (m, 4H), 2.26–2.19 (m, 2H), 2.18–2.13 (m, 2H), 2.08–1.98 (m, 2H), 1.99–1.90 (m, 2H), 1.88–1.80 (m, 5H), 1.71 (m, 2H), 1.62 (m, 1H), 1.09–1.07 (m, 1H), 1.07–1.04 (m, 1H). ^13^C-NMR (151 MHz, Chloroform-*d*) δ 165.94, 142.17, 133.41, 133.15, 127.74, 127.67, 126.91, 63.98, 55.87, 55.80, 55.74, 47.68, 42.76, 31.93, 26.29, 24.65, 23.91, 22.74, 21.57, 21.36. MS (ESI) *m*/*z*: 377.487 [M + H]^+^.

**2j** Yield: 37%, m.p.: 96.5–97.7 °C; Yellow solid; ^1^H-NMR (600 MHz, Chloroform-*d*) δ 7.83 (d, *J* = 1.9 Hz, 1H), 7.06 (d, *J* = 3.6 Hz, 1H), 6.76 (m, 1H), 3.51 (s, 1H), 2.91 (m, 4H), 2.53 (m, 3H), 2.48 (d, *J* = 1.0 Hz, 1H), 2.30–2.13 (m, 1H), 2.03 (m, 1H), 1.61 (m, 5H), 1.29 (m, 7H), 1.19–1.10 (m, 1H), 0.90 (t, *J* = 6.9 Hz, 1H). ^13^C-NMR (151 MHz, Chloroform-*d*) δ 165.15, 143.26, 137.33, 131.69, 129.92, 127.62, 125.68, 55.97, 47.97, 35.93, 31.93, 29.78, 29.70, 29.33, 29.25, 27.22, 26.78, 23.80, 22.69, 15.53, 14.13. MS (ESI) *m*/*z*: 357.510 [M + H]^+^.

**2k** Yield: 44%, m.p.:119.6–122.4 °C; White solid; ^1^H-NMR (600 MHz, Chloroform-*d*) δ 7.99 (d, *J* = 1.9 Hz, 1H), 7.35 (d, *J* = 5.1 Hz, 1H), 6.94 (d, *J* = 5.1 Hz, 1H), 3.64 (dd, *J* = 13.7, 5.1 Hz, 1H), 3.57–3.50 (m, 1H), 3.35 (t, *J* = 12.7 Hz, 1H), 3.02 (m, 1H), 2.96–2.79 (m, 2H), 2.68–2.59 (m, 1H), 2.39 (s, 3H), 2.27–0.70 (m, 15H). ^13^C-NMR (151 MHz, Chloroform-*d*) δ 165.20, 140.32, 132.71, 130.24, 126.29, 126.00, 125.53, 63.52, 56.06, 55.24, 50.64, 48.03, 39.90, 31.51, 30.14, 26.91, 23.82, 22.70, 21.56, 21.51, 14.61. MS (ESI) *m*/*z*: 357.481 [M + H]^+^.

**3a** Yield: 40%, m.p.: 82.9–83.4 °C; Yellow solid; ^1^H-NMR (600 MHz, Chloroform-*d*) δ 3.47–3.43 (m, 1H), 3.39–3.34 (m, 1H), 3.30–3.09 (m, 1H), 2.91–2.83 (m, 1H), 2.76 (m, 1H), 2.17–2.08 (m, 3H), 2.05 (m, 1H), 2.01–1.96 (m, 1H), 1.95–1.85 (m, 4H), 1.82–1.75 (m, 1H), 1.74–1.59 (m, 2H), 1.56–1.43 (m, 4H), 1.43–1.35 (m, 1H), 1.33–1.24 (m, 1H), 1.11–1.00 (m, 1H), 0.93 (m, 4H). ^13^C-NMR (151 MHz, Chloroform-*d*) δ 172.52, 64.02, 56.09, 55.59, 50.84, 47.61, 42.96, 41.61, 32.08, 29.81, 27.79, 24.56, 24.24, 24.05, 21.35, 21.04, 11.43. MS (ESI) *m*/*z*: 277.317 [M + H]^+^.

**3b** Yield: 44%, m.p.: 71.1–71.8 °C; Yellow solid; ^1^H-NMR (600 MHz, Chloroform-*d*) δ 3.44 (m, 1H), 3.17–3.11 (m, 1H), 2.86 (m, 1H), 2.78–2.74 (m, 1H), 2.23 (m, 2H), 2.12 (m, 2H), 2.06–2.02 (m, 1H), 1.81–1.76 (m, 2H), 1.73 (m, 2H), 1.60 (m, 2H), 1.53–1.48 (m, 4H), 1.40 (m, 3H), 1.32–1.28 (m, 4H), 1.05 (m, 1H), 0.89 (m, 5H). ^13^C-NMR (151 MHz, Chloroform-*d*) δ 172.69, 63.99, 62.54, 56.09, 55.57, 50.82, 47.64, 41.58, 41.22, 32.03, 30.61, 29.84, 29.28, 24.61, 22.79, 22.20, 21.38, 21.07, 14.06. MS (ESI) *m*/*z*: 305.436 [M + H]^+^.

**3c** Yield: 61%, m.p.: 68.1–68.8 °C; Yellow solid; ^1^H-NMR (600 MHz, Chloroform-*d*) δ 3.47–3.42 (m, 1H), 3.15 (d, *J* = 12.3 Hz, 1H), 2.85 (m, 2H), 2.77–2.72 (m, 2H), 2.29–2.20 (m, 2H), 2.16–2.10 (m, 2H), 2.05 (m, 1H), 1.79 (m, 2H), 1.77–1.72 (m, 1H), 1.38–1.34 (m, 1H), 1.33–1.26 (m, 13H), 1.10–1.00 (m, 2H), 0.87 (m, 6H). ^13^C-NMR (151 MHz, Chloroform-*d*) δ 172.69, 63.99, 56.09, 55.57, 50.82, 47.64, 41.65, 41.25, 31.79, 29.36, 27.03, 24.68, 24.62, 24.22, 23.10, 22.63, 22.20, 21.84, 21.39, 21.07, 14.08. MS (ESI) *m*/*z*: 333.398 [M + H]^+^.

**3d** Yield: 42%, m.p.: 163.5–165.7 °C; White solid; ^1^H-NMR (600 MHz, Chloroform-*d*) δ 7.28 (dd, *J* = 8.2, 6.9 Hz, 2H), 7.25–7.16 (m, 3H), 3.50 (m, 2H), 3.37 (m, 1H), 3.23–3.12 (m, 1H), 2.87 (m, 1H), 2.77 (m, 1H), 2.60 (m, 1H), 2.47 (m, 1H), 2.30–2.13 (m, 1H), 2.13–2.00 (m, 2H), 1.91 (m, 3H), 1.82 (m, 1H), 1.71 (m, 3H), 1.67–1.57 (m, 1H), 1.58–1.49 (m, 1H), 1.46 (m, 2H), 1.37–1.29 (m, 1H), 1.24 (m, 1H), 1.06 (m, 1H). ^13^C-NMR (151 MHz, Chloroform-*d*) δ 171.71, 140.32, 129.28 (2), 128.28 (2), 126.00, 64.00, 56.08, 55.68, 50.82, 47.84, 43.57, 41.72, 37.80, 32.05, 29.81, 27.86, 24.26, 24.25, 21.29, 20.94. MS (ESI) *m*/*z*: 339.382 [M + H]^+^.

**3e** Yield: 53%, m.p.: 110.1–112.5 °C; Yellow solid; ^1^H-NMR (600 MHz, Chloroform-*d*) δ 7.12 (dd, *J* = 5.1, 1.2 Hz, 1H), 6.91 (dd, *J* = 5.2, 3.4 Hz, 1H), 6.80 (dd, *J* = 3.5, 1.0 Hz, 1H), 3.49 (m, 2H), 3.34 (m, 1H), 3.19–3.10 (m, 1H), 3.05 (m, 1H), 2.86 (m, 1H), 2.75 (m, 1H), 2.65–2.46 (m, 2H), 2.31–2.21 (m, 1H), 2.16–2.08 (m, 2H), 2.06–1.97 (m, 1H), 1.90 (m, 3H), 1.84–1.76 (m, 1H), 1.70 (m, 2H), 1.61 (m, 1H), 1.56–1.42 (m, 3H), 1.30–1.23 (m, 1H), 1.13–0.99 (m, 1H). ^13^C-NMR (151 MHz, Chloroform-*d*) δ 171.23, 154.31, 141.04, 110.18, 106.41, 64.05, 56.10, 55.67, 50.87, 47.81, 41.81, 41.52, 32.11, 30.13, 29.78, 28.02, 24.79, 24.30, 21.28, 20.90. MS (ESI) *m*/*z*: 329.363 [M + H]^+^.

**3f** Yield: 33%; Brown oil; ^1^H-NMR (600 MHz, Chloroform-*d*) δ 7.32 (m, 1H), 6.30 (m, 1H), 6.05 (m, 1H), 3.51 (m, 1H), 3.49–3.34 (m, 2H), 3.33–3.25 (m, 1H), 3.24–3.12 (m, 1H), 2.87 (m, 1H), 2.85–2.72 (m, 2H), 2.65–2.53 (m, 1H), 2.31–2.21 (m, 1H), 2.12 (m, 2H), 2.08–1.99 (m, 1H), 1.98–1.90 (m, 2H), 1.90–1.82 (m, 2H), 1.80–1.56 (m, 3H), 1.56–1.44 (m, 2H), 1.44–1.32 (m, 1H), 1.31–1.24 (m, 1H), 1.16–1.02 (m, 1H). ^13^C-NMR (151 MHz, Chloroform-*d*) δ 171.14, 142.36, 126.60, 125.80, 123.70, 64.03, 56.07, 55.64, 50.84, 47.86, 43.63, 41.79, 32.10, 31.90, 29.72, 28.03, 24.33, 24.26, 21.20, 20.84. MS (ESI) *m*/*z*: 345.417 [M + H]^+^.

### 3.4. Anti-Proliferation Assay

The HepG-2 (human liver cancer cell line) and CNE-2 (human nasopharyngeal carcinoma cell line) were obtained from American Type Culture Collection (ATCC). The suspension (100 μL/well) with evaluated cells (3~4 × 10^4^ cell/mL) and DMEM culture medium of 10% foetal bovine serum (FBS) was seeded into 96-well plates. After a 24 h incubation period in 5% CO_2_, media was replaced with solution of different concentrations (5, 10, 20, 50, 100 μM) of compounds. The solution was made by serial dilution in culture medium (DMEM of 10% foetal bovine serum) of stock solutions of test compounds prepared in DMSO. Final concentration of DMSO was less than 0.1% in each well. Cells were cultured for 48 h, then 20 μL of 5 mg/mL MTT ([3-(4,5-dimethylthiazol-2-yl)-2,5-diphenyltetrazolium bromide]) were added to each well, followed by incubation for 4 h at 37 °C. The supernatants were removed and 150 μL DMSO was added to each well for the colorimetric reaction. Finally, the optical density was measured at the 490 nm wavelength on an enzyme-linked immunosorbent assay microplate reader.

### 3.5. Cell Cycle Analysis

HepG2 cells were treated with **2k** at concentrations of 10, 20, and 30 μmol/L for 24 h and then cells were harvested. Cells were fixed with 75% ethanol at 4 °C overnight. The fixed cells were incubated with 100 mg/mL RNase at 37 °C for 30 min and then stained with 50 mg/mL propidium iodide in the dark for 30 min. Cell cycle distribution was then analyzed by flow cytometry using FACS analysis.

### 3.6. Molecular Docking

Molecular docking studies were performed using the Glide model from Schrödinger software. The structure of the compounds was sketched using ChemDraw and optimized to lower energy conformers using Ligprep (Schrodinger LLC, New York, NY, USA). The structure of human DNA–Topo I complex (PBD ID: 1T8I) was downloaded from the Protein Data Bank and prepared for docking using protein preparation wizard. After a series of preprocessing, such as mutation, adding hydrogens, deleting water etc., OPLS3 force field was used for optimizing the hydrogen bond network in the enzyme structure. Then minimization was carried out until the energy converged or the route mean square deviation (RMSD) reached a maximum cutoff of 0.30 Å. The receptor grid can be set up and generated from the Receptor Grid Generation panel. Ultimately, the compounds were performed to dock on generated grid of protein structure using Ligand Docking under the XP (extra precision) precision. Glide (docking) score is the evaluation standard of ligand-protein binding.

## 4. Conclusions

In conclusion, a series of α, β-unsaturated sophoridinal derivatives have been successfully designed, synthesized, and characterized. The preliminary biological screening of the synthesized compounds indicated that **2e** and **2k** exhibited potent anti-proliferative activities against hepatocellular carcinoma and nasopharyngeal carcinoma. Besides, SAR analysis revealed that the formation of α, β-unsaturated ketone could enhance the activity effectively and verified the rationality of the design. The introduced aryl and heterocyclic rings could increase binding affinity with purine ring of DNA through π–π stacking interaction. Moreover, decorating various substituents on the ring played an important role in drug like physio–chemical properties of the compounds. Based on the cell cycle analysis results, it is suggested that the mode of action is to inhibit the activity of DNA topo I, followed by the G0/G1 phase arrest. This work provides useful information for further structural modifications of these compounds and for the synthesis of new, potent antitumor agents.

## Figures and Tables

**Table 1:**
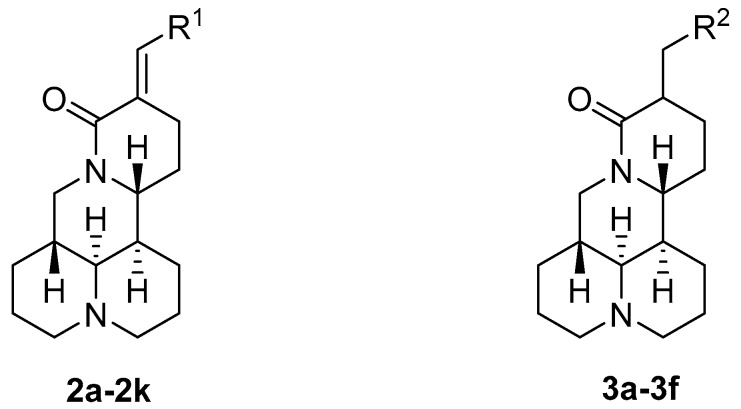
Structure-activity relationship of the sophoridinic derivatives for their antiproliferative activities in HepG-2 (human liver cancer cell line) and CNE-2 (human nasopharyngeal carcinoma cell line) cells.

Compounds	R^1^	R^2^	IC_50_ (μM)		Clog P ^a^
			HepG-2	CNE-2	
Sophoridine			4670 ± 127	5379 ± 109	1.36
**2a**	CH_2_CH_2_CH_2_CH_3_	/	82.3 ± 9.3	>100	3.99
**2b**	Ph	/	>100	87.5 ± 6.8	3.73
**2c**	PhOCH_3_-p	/	>100	>100	3.65
**2d**	PhCl-p	/	55.7 ± 5.6	>100	4.44
**2e**	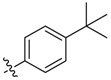	/	35.1 ± 2.9	38.3 ± 2.1	5.56
**2f**	3-naphthyl	/	75.5 ± 8.3	95.3 ± 4.8	4.9
**2g**	2-furyl	/	92.3 ± 7.1	89.5 ± 8.3	2.91
**2h**	2-thienyl	/	>100	>100	3.38
**2i**	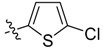	/	96.4 ± 5.7	89.4 ± 7.8	4.13
**2j**	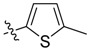	/	>100	>100	3.87
**2k**	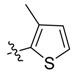	/	25.5 ± 3.3	33.8 ± 2.3	3.87
**3a**	/	CH_3_	>100	>100	2.41
**3b**	/	CH_2_CH_2_CH_3_	>100	>100	3.47
**3c**	/	CH_2_CH_2_CH_2_CH_2_CH_3_	57.9 ± 6.3	83.1 ± 9.2	4.53
**3d**	/	Ph	>100	>100	3.45
**3e**	/	2-furyl	>100	>100	2.62
**3f**	/	2-thienyl	>100	>100	3.09
Taxo			15.6 ± 2.3	0.18	

^a^ Lipophilicity of the compounds is expressed in terms of their Clog P values that were calculated using ChemBioDraw Ultra 12.0 software.
